# Presumed Paravertebral Inflammatory Pseudotumor in Relapsing Granulomatosis With Polyangiitis: Fluorodeoxyglucose (FDG) Positron Emission Tomography/Computed Tomography (PET/CT) Findings and Complete Metabolic Response to Rituximab-Based Therapy

**DOI:** 10.7759/cureus.117516

**Published:** 2026-10-05

**Authors:** Yasmina Hamida El Khloufi, Redouane Soussi, Pascale Bohy

**Affiliations:** 1 Radiology, Erasmus Hospital, Bruxelles, BEL; 2 Nuclear Medicine, Erasmus Hospital, Bruxelles, BEL

**Keywords:** anca-associated vasculitis, fdg pet/ct, granulomatosis with polyangiitis, inflammatory pseudotumor, paravertebral mass, rituximab

## Abstract

We report a case of a 60-year-old woman with biopsy-proven, proteinase-3 (PR3)-antineutrophil cytoplasmic antibody (ANCA)-associated granulomatosis with polyangiitis (GPA), diagnosed six years earlier, who presented with right lower thoracic pain during a disease relapse. The relapse was supported by serological reactivation with a markedly elevated anti-PR3 level, a raised erythrocyte sedimentation rate (ESR) (88 mm/h), and elevated C-reactive protein (CRP) (48 mg/L). Unenhanced computed tomography (CT) showed no pulmonary parenchymal abnormality but revealed soft-tissue infiltration of the anterior and right lateral paravertebral mediastinal fat extending from T4-T5 to T10-T11, without adjacent osseous involvement. Fluorine-18 (^18^F)-fluorodeoxyglucose (FDG) positron emission tomography/CT (PET/CT) demonstrated intense uptake within the lesion, with a maximum standardized uptake value (SUVmax) of 12.06. Given the differential diagnoses of lymphoma and other granulomatous disease, the lesion was interpreted as a presumed GPA-related paravertebral inflammatory pseudotumor; it was not biopsied. After rituximab and methylprednisolone, the patient achieved complete clinical and biological remission with ANCA seroconversion, and follow-up FDG PET/CT at three months showed complete resolution of the abnormal uptake. Because neither FDG avidity nor treatment response was specific, the presumed diagnosis could not be confirmed histologically in this case.

## Introduction

Granulomatosis with polyangiitis (GPA), formerly known as Wegener’s granulomatosis, is a rare systemic necrotizing small-vessel vasculitis classified among the antineutrophil cytoplasmic antibody (ANCA)-associated vasculitides. It classically presents with a triad of upper respiratory tract, lower respiratory tract, and renal involvement, and pulmonary parenchymal disease is present in the majority of patients [1].

Mass-forming or “pseudotumoral” lesions are an uncommon manifestation of GPA. Histologically, they correspond to granulomatous inflammation and only rarely to overt vasculitis. They have been described in the orbit, paranasal sinuses, ear, nose, and throat (ENT) region, mediastinum, breast, kidney, and retroperitoneum, where they may mimic malignancy, infection, or fibrosing mediastinitis [1,2]. Paravertebral localization is rare, with only a limited number of reported cases [2-4]. These lesions may occur at disease onset or during follow-up, are frequently not associated with bone erosion, and may be asymptomatic or produce atypical thoracic or back pain [2-4].

We report an atypical case of relapsing GPA with a presumed paravertebral pseudotumoral mediastinal manifestation in the absence of pulmonary parenchymal disease, characterized by computed tomography (CT) and fluorine-18 (^18^F)-fluorodeoxyglucose (FDG) positron emission tomography/CT (PET/CT), with a complete clinical and metabolic response to rituximab-based therapy.

## Case presentation

A 60-year-old woman had a six-year history of biopsy-proven, proteinase-3 (PR3)-ANCA-associated GPA. The disease was initially diagnosed during a hospitalization for multisystem involvement and was confirmed by a biopsy of the Eustachian tube mucosa showing granulomas and necrosis, in association with cytoplasmic-pattern ANCA (c-ANCA) of PR3 specificity. At diagnosis, the biological workup showed a marked inflammatory syndrome, comprising an erythrocyte sedimentation rate (ESR) of 92 mm/h, C-reactive protein (CRP) of 37 mg/L, and hyperfibrinogenemia, with c-ANCA at a titer of 1:160 (anti-PR3 9.8 U/mL, reference range < 2.0 U/mL), negative anti-myeloperoxidase and antinuclear antibodies, a polyclonal inflammatory pattern without a monoclonal component on serum protein electrophoresis, preserved renal function (serum creatinine 0.6 mg/dL; estimated glomerular filtration rate > 60 mL/min/1.73 m²), and a negative infectious serological screen (hepatitis B and C, HIV, cytomegalovirus, Epstein-Barr virus, syphilis, and Toxoplasma). The initial disease was multisystemic, with a Birmingham Vasculitis Activity Score for Wegener granulomatosis (BVAS/WG) [5] of 6, and comprised otological involvement (bilateral middle-ear effusion requiring tympanostomy tubes, with necrotic lesions of the left Eustachian tube), neurological involvement (pachymeningitis and a right hypoglossal nerve palsy related to a hypermetabolic retropharyngeal and pericarotid infiltrate producing extrinsic-appearing stenosis of both carotid arteries), and an FDG-avid infiltrate of the left upper pulmonary lobe. Remission was obtained with pulsed cyclophosphamide and corticosteroids.

Six years later, the patient presented with right lower thoracic (basi-thoracic) pain. Laboratory evaluation showed a recurrent inflammatory syndrome (ESR 88 mm/h; CRP 48 mg/L) with mild normocytic anemia (hemoglobin 11.6 g/dL). ANCA testing was positive, with a cytoplasmic-pattern immunofluorescence titer of 1:20 and a markedly elevated anti-PR3 antibody level (523 U/mL; reference range < 20 U/mL), indicating serological reactivation. The diagnosis of relapse was based on the combination of new clinical symptoms (right lower thoracic pain), a recurrent inflammatory syndrome, new imaging findings (described below), and the rise in PR3-ANCA, rather than on the serological change alone; ANCA titers may correlate imperfectly with disease activity, and an isolated serological rise does not by itself establish relapse (Table 1).

**Table 1:** Laboratory findings at diagnosis and at relapse (six years later) ^a^Anti-PR3 was measured at diagnosis and relapse using different assays, with reference ranges < 2.0 U/mL and < 20 U/mL, respectively; therefore, the two values are not directly comparable. Reference ranges are those of the reporting laboratory and differed slightly between the two time points. Dashes indicate tests not repeated at relapse. c-ANCA: cytoplasmic-pattern antineutrophil cytoplasmic antibody; CKD-EPI: Chronic Kidney Disease Epidemiology Collaboration; GFR: glomerular filtration rate

Test	At diagnosis	At relapse	Reference range	Unit
Erythrocyte sedimentation rate (ESR)	92	88	< 20	mm/h
C-reactive protein (CRP)	37	48	< 5	mg/L
Fibrinogen	494	-	160-400	mg/dL
Hemoglobin	12.8	11.6	12-16	g/dL
Lymphocytes (absolute)	1.24	1.36	1.2-3.5	×10³/mm³
Platelets	359	416	150-440	×10³/mm³
Serum creatinine	0.6	-	0.5-1.0	mg/dL
Estimated GFR (CKD-EPI)	105	-	> 60	mL/min/1.73 m²
c-ANCA (indirect immunofluorescence)	1:160	1:20	Negative	titer
Anti-proteinase-3 (PR3) antibodyᵃ	9.8	523	See footnote	U/mL
Anti-myeloperoxidase (MPO) antibody	< 1.0	-	< 3.5	U/mL
Antinuclear antibodies (ANA)	Negative	-	Negative	-
Serum protein electrophoresis	Polyclonal; no monoclonal component	-	-	-

Unenhanced thoracoabdominal CT showed no pulmonary nodules, cavitation, consolidation, or other parenchymal abnormality suggestive of GPA. It did, however, reveal ill-defined soft-tissue infiltration of the mediastinal fat in the anterior and right lateral paravertebral compartment, extending craniocaudally from the T4-T5 to the T10-T11 level (Figure 1). The infiltrate measured 30 mm transversely, 12 mm anteroposteriorly, and 130 mm craniocaudally, with soft-tissue attenuation values of 45-50 Hounsfield units. It abutted the right margin of the descending thoracic aorta and the azygos vein without displacing them; as the study was performed without intravenous contrast, vascular involvement could not be further characterized. No adjacent osseous erosion or destruction was identified. In the context of known GPA, the appearance was considered most consistent with a paravertebral inflammatory pseudotumor; lymphoma and granulomatous disease of another etiology were retained as differential diagnoses.

**Figure 1:**
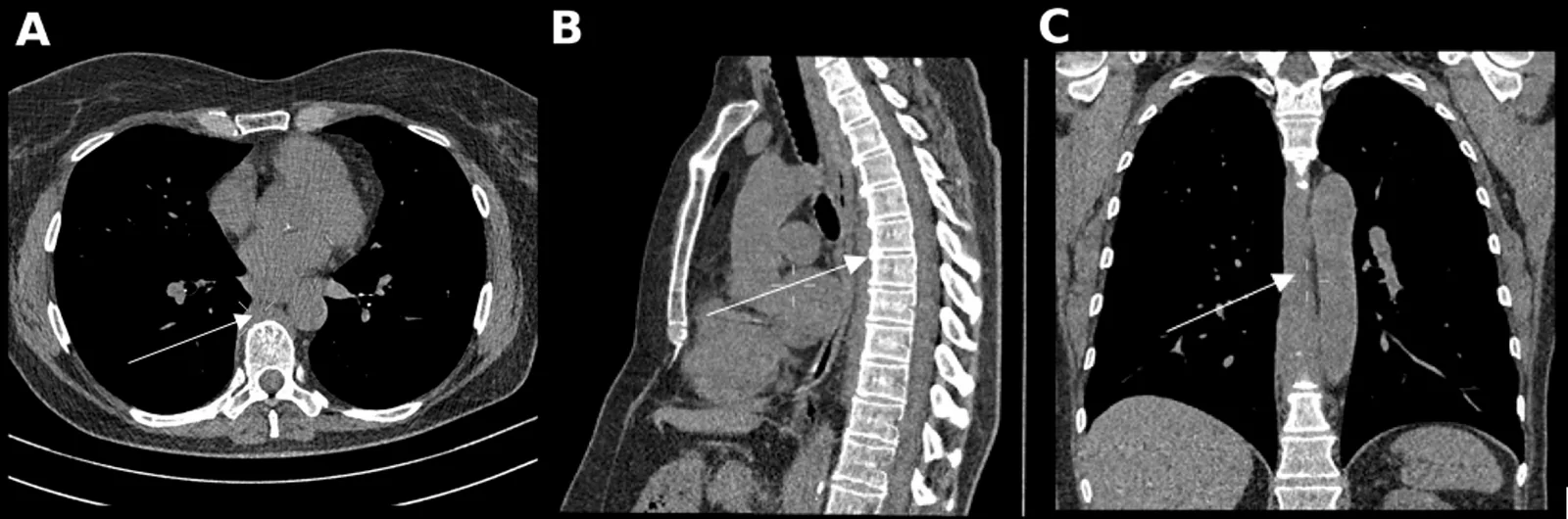
Unenhanced thoracic CT Axial (A), sagittal (B), and coronal (C) images showing ill-defined soft-tissue infiltration of the anterior and right lateral paravertebral mediastinal fat extending from the T4-T5 to the T10-T11 level and abutting the right margin of the descending thoracic aorta and the azygos vein, without adjacent osseous involvement. The arrows indicate the paravertebral soft-tissue infiltration. CT: computed tomography

Given the established, biopsy-proven diagnosis of PR3-ANCA GPA, the strongly positive serology, the concordant inflammatory syndrome, and the patient’s prior propensity for FDG-avid mass-like inflammatory lesions, the paravertebral lesion was considered most consistent with a relapse-related GPA pseudotumoral manifestation. Image-guided percutaneous biopsy of the paravertebral mass was considered by the radiologist but not performed. Given the previously established histological diagnosis of PR3-ANCA GPA and the concordant clinical, serological, biological, and imaging picture of relapse, a multidisciplinary team decision was made not to biopsy the lesion and to treat and reassess by imaging instead. The differential diagnoses of lymphoma, infection, IgG4-related disease, and other granulomatous disorders were nonetheless kept in mind, and the interpretation was revisited in light of the complete response to therapy. The subsequent complete clinical and metabolic response was consistent with, but did not confirm, this interpretation; in the absence of histology, the lesion is reported as a presumed GPA-related manifestation.

^18^F-FDG PET/CT demonstrated intense focal hypermetabolism corresponding to the paravertebral mass, with a maximum standardized uptake value (SUVmax) of 12.06 and a metabolic tumor volume of 43.78 mL (Figure 2), in keeping with a metabolically active lesion. An additional hypermetabolic focus was present in the left presacral region, posterior to the left common iliac vessels, on the whole-body FDG PET/CT (SUVmax 14.2; metabolic tumor volume 14.78 mL). As the dedicated diagnostic CT was limited to the thorax, this focus was characterized on the low-dose PET/CT only and had no dedicated contrast-enhanced CT correlate; it was therefore interpreted as a presumed further site of active GPA, on the basis of its FDG avidity concordant with the paravertebral lesion and its complete resolution after immunosuppressive therapy, while acknowledging that this attribution, like that of the paravertebral lesion, was not histologically confirmed. The metabolic findings, although unable to distinguish inflammatory from neoplastic tissue, were consistent with active GPA.

**Figure 2:**
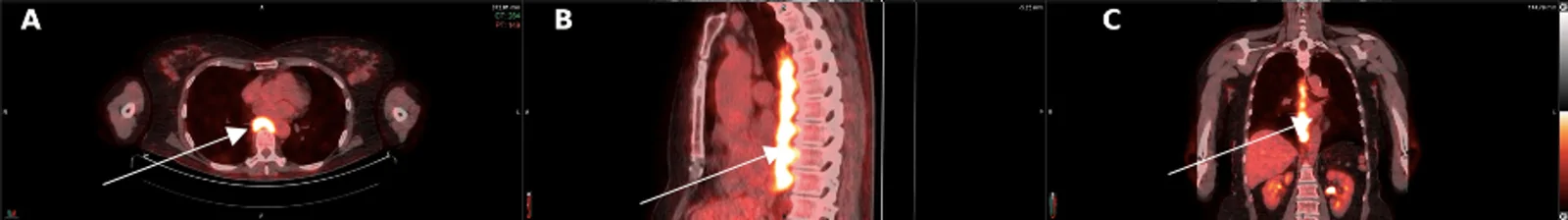
Baseline 18F-FDG PET/CT Fused axial (A), sagittal (B), and coronal (C) images showing intense hypermetabolism (SUVmax 12.06; metabolic tumor volume 43.78 mL) along the elongated paravertebral lesion. An additional hypermetabolic focus in the left presacral region, posterior to the left common iliac vessels (SUVmax 14.2; metabolic tumor volume 14.78 mL), was also identified. The arrows indicate the FDG-avid paravertebral lesion. ^18^F: fluorine-18; FDG: fluorodeoxyglucose; PET: positron emission tomography; CT: computed tomography; SUVmax: maximum standardized uptake value

Remission induction consisted of rituximab 375 mg/m² intravenously once weekly for four infusions, together with methylprednisolone on a tapering oral regimen (24 mg/day from week 2, 20 mg/day at weeks 3-4, with subsequent progressive tapering). Prophylaxis against *Pneumocystis jirovecii *with trimethoprim-sulfamethoxazole (800/160 mg every other day) was also given. Thoracic pain resolved, inflammatory markers normalized, and ANCA became negative. A follow-up ^18^F-FDG PET/CT performed three months after treatment initiation was completely negative, demonstrating resolution of the previously abnormal metabolic activity which, together with the clinical and laboratory course, was consistent with complete remission (Figure 3). At subsequent six-monthly clinical and biological surveillance, she has remained in complete remission.

**Figure 3:**
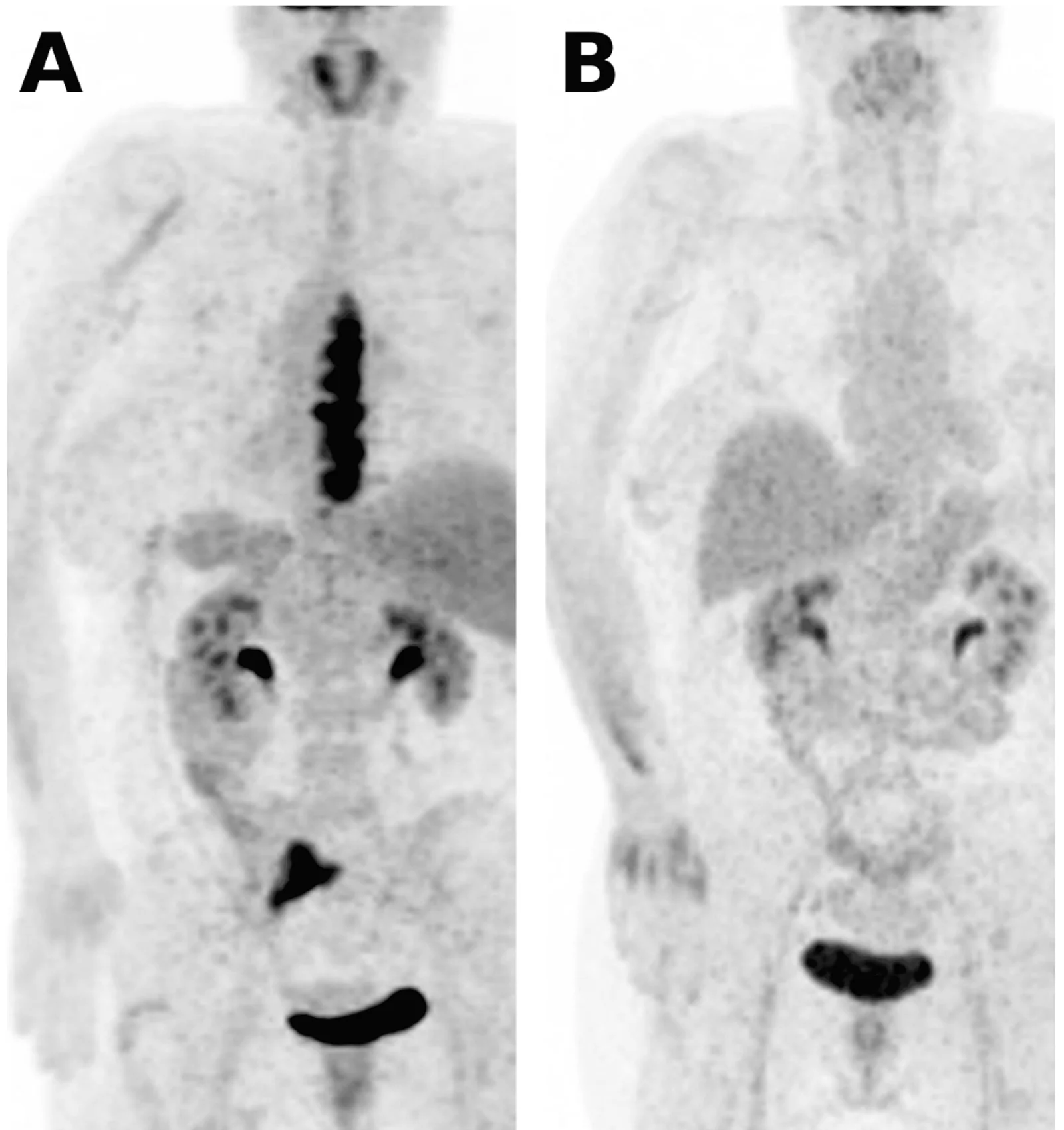
Metabolic response on 18F-FDG PET/CT Maximum-intensity-projection ^18^F-FDG PET/CT images (A) at baseline and (B) three months after rituximab and corticosteroid therapy, showing complete disappearance of the paravertebral uptake at follow-up (B), consistent with a complete metabolic response. ^18^F: fluorine-18; FDG: fluorodeoxyglucose; PET: positron emission tomography; CT: computed tomography

## Discussion

GPA is a rare ANCA-associated small-vessel vasculitis in which the respiratory tract and kidneys are the most commonly affected organs, and pulmonary involvement is reported in the large majority of patients [1]. The present case is atypical in two respects: the disease manifested as a paravertebral pseudotumoral mass, and it did so in the absence of any parenchymal lung disease.

Tumor-like mass lesions are an uncommon and diagnostically challenging manifestation of GPA. They may arise as a localized feature or as part of systemic disease, show granulomatous inflammation on histology, and have been reported in numerous sites including the mediastinum [2,6,7]. Paravertebral (dorsal prevertebral) lesions are particularly rare; small series and case reports have described them as frequently free of bone erosion, sometimes asymptomatic, and occasionally responsible for chronic or atypical thoracic pain [2-4]. Our patient’s presentation, with a right-sided paravertebral soft-tissue infiltration extending over several thoracic levels without osseous involvement and manifesting as basi-thoracic pain, is concordant with these previous descriptions. Of note, this patient had already shown a propensity for FDG-avid, mass-like lesions at unusual sites during her initial flare, including a retropharyngeal and pericarotid infiltrate and a left upper-lobe pulmonary infiltrate. The new paravertebral lesion, together with the concurrent soft-tissue focus anterior to the left iliac vessels, therefore occurred within a previously documented infiltrative, often perivascular, pseudotumoral phenotype of her GPA, strengthening the clinicoradiological attribution to disease relapse.

The principal diagnostic difficulty lies in distinguishing a GPA pseudotumor from malignancy - chiefly lymphoma - as well as from fibrosing mediastinitis, infection (including tuberculosis and fungal disease), sarcoidosis, histiocytosis, and IgG4-related disease [2,4,7]. Because a mediastinal or paravertebral mass may be the presenting or dominant feature, GPA can be mistaken for a neoplastic process, and histological confirmation is often required. When technically feasible and diagnostically necessary, image-guided biopsy can establish the nature of the lesion; reported samples typically show granulomatous inflammation and only rarely frank vasculitis [2-4]. In the present case, no biopsy of the paravertebral lesion was obtained. The attribution to GPA rested on the combination of biopsy-proven PR3-ANCA GPA, marked serological and inflammatory reactivation, a prior history of unusual FDG-avid mass-like inflammatory lesions, the simultaneous presence of another active soft-tissue focus, and complete clinical and imaging remission after immunosuppressive therapy. A polyclonal serum electrophoretic pattern and negative infectious investigations provided additional contextual information but did not, in isolation, exclude lymphoma or other alternative diagnoses.

FDG PET/CT contributes complementary information. In GPA, active lesions are typically intensely FDG-avid, and PET/CT can reveal occult sites of disease, help select the optimal biopsy site, and provide an objective measure of treatment response through a reduction in metabolic activity [8,9]. Importantly, FDG uptake intensity does not reliably differentiate inflammatory GPA lesions from malignancy, so a metabolically active mass cannot be assumed to be benign on PET criteria alone [8]. In our patient, the intense uptake confirmed an active lesion and supported both disease mapping and subsequent response assessment, while underscoring why malignancy had to be formally considered.

Rituximab, an anti-CD20 monoclonal antibody, in combination with glucocorticoids, is an established first-line option for remission induction in severe GPA, has been shown to be non-inferior to cyclophosphamide, and is frequently preferred in relapsing disease [10,11]. Pseudotumoral GPA lesions generally regress with immunosuppressive therapy, whereas long-standing, fibrotic lesions may remain stable [2]. In our patient, thoracic pain, inflammatory abnormalities, ANCA positivity, and all abnormal FDG uptake resolved after rituximab and methylprednisolone, which was concordant with successful treatment of active GPA. However, because rituximab and glucocorticoids can also induce regression of certain lymphoid neoplasms, therapeutic response cannot be regarded as specific proof of the nature of an unbiopsied mass.

This report has limitations inherent to a single case. Most importantly, the paravertebral lesion was not biopsied, so its attribution to GPA remains clinicoradiological rather than histologically proven. In addition, complete regression after rituximab and glucocorticoids, although highly concordant with the overall picture of active GPA, is not diagnostically specific, because some lymphoproliferative disorders may also respond to these agents. The imaging and laboratory data should therefore be interpreted within the full clinical context. Nevertheless, the combination of biopsy-proven PR3-ANCA GPA, marked relapse activity, a previously documented pseudotumoral/infiltrative disease phenotype, and complete metabolic remission supports the proposed diagnosis and adds to the small body of literature on paravertebral pseudotumoral manifestations of GPA.

## Conclusions

Relapsing GPA may present with a paravertebral inflammatory pseudotumoral lesion in the absence of pulmonary parenchymal disease, and such lesions can be intensely FDG-avid and mimic malignancy. FDG PET/CT is valuable for mapping active disease and documenting metabolic response. Because neither FDG avidity nor regression after immunosuppression is diagnostically specific, integration of clinical history, serological activity, imaging, and, when feasible, histological data remains essential for accurate diagnosis and appropriate management.
